# Life Stage‐ and Sex‐Specific Sensitivity to Nutritional Stress in a Holometabolous Insect

**DOI:** 10.1002/ece3.70764

**Published:** 2025-01-18

**Authors:** Leon Brueggemann, Pragya Singh, Caroline Müller

**Affiliations:** ^1^ Department of Chemical Ecology Bielefeld University Bielefeld Germany; ^2^ Joint Institute for Individualisation in a Changing Environment (JICE) University of Münster and Bielefeld University Bielefeld Germany

**Keywords:** behaviour, energy metabolism, life‐history, niche conformance, phenotypic plasticity, sensitive phases, starvation

## Abstract

Over lifetime, organisms can be repeatedly exposed to stress, shaping their phenotype. At certain, so‐called sensitive phases, individuals might be more receptive to such stress, for example, nutritional stress. However, little is known about how plastic responses differ between individuals experiencing nutritional stress early versus later in life or repeatedly, particularly in species with distinct ontogenetic niches. Moreover, there may be sex‐specific differences due to distinct physiology. Larvae of the holometabolous turnip sawfly, *Athalia rosa*e, consume leaves and flowers, while the adults take up nectar. We examined the effects of starvation experienced at different life stages on life‐history, adult behavioural and metabolic traits to determine which stage may be more sensitive and how specific these traits respond. We exposed individuals to four nutritional regimes, either no, larval, adult starvation or starvation periods as larvae and adults. Larvae exposed to starvation had a prolonged development, and starved females reached a lower initial adult body mass than non‐starved individuals. Males did not differ in initial adult body mass regardless of larval starvation, suggesting the ability to conform well to poor nutritional conditions. Adult behavioural activity was not significantly impacted by larval or adult starvation. Individuals starved as larvae had similar carbohydrate and lipid (i.e., fatty acid) contents as non‐starved individuals, potentially due to building up energy reserves during development, while starvation during adulthood or at both stages led to reduced energy reserves in males. This study indicates that the sensitivity of a life stage to stress depends on the specific trait under consideration. Life‐history traits were mainly affected by larval stress, while activity appeared to be more robust and metabolism mostly impacted by the adult conditions. Individuals differed in their ability to conform to the given environment, with the responses being life stage‐ and sex‐specific.

## Graphical abstract

Our study provides novel insights into trait‐specific sensitivity to nutritional stress across sex and life stages. By integrating life‐history, behaviour and metabolism, we explore mechanisms on an individual level, offering a comprehensive view of how these traits influence ecological patterns across ontogenetic niches of a holometabolous insect.

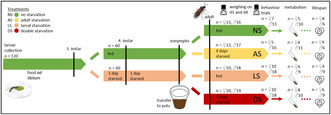

## 1 Introduction

Sensitive phases are periods during ontogeny when environmental cues, such as temperature, humidity and resource availability, profoundly influence development and subsequent life‐history strategies (Fawcett and Frankenhuis 2015; Walasek et al. 2024). These phases critically shape an individual's morphology, behaviour and physiology (Macdonald 1985; Monaghan 2008). Determined by the genetic architecture and plasticity of the individual, sensitive phases are documented across different life stages and diverse taxa, including birds, rodents and humans (Oyama 1979; Baptista and Petrinovich 1984; Sachser et al. 2020). In organisms that undergo distinct life stages, such as holometabolous insects, sensitive phases may be particularly important, because individual responses to stressors and their general resource requirements are likely to differ (English and Barreaux 2020). For example, in several insect species larvae feed on leaves, while adults feed on nectar; thus, distinct stages show ontogenetic niche shifts (Dopman, Sword, and Hillis 2002). Investigating sensitive phases in holometabolous insects may help us understand population dynamics and evolutionary trajectories under environmental change scenarios and help in developing pest management and conservation strategies (Frankenhuis, Nettle, and Dall 2019; Smith et al. 2020).

In nature, individuals may face different stresses throughout their lifespans, with potential for additive effects (Todgham and Stillman 2013). In response to stressful environments, individuals can alter their phenotype, such as life‐history traits, behaviour and the metabolism, leading to niche conformance (Müller et al. 2020). Moreover, individuals may respond differently, leading to individualised niches (Trappes et al. 2022; Kaiser et al. 2024). Recurrent stress exposure across life stages is less studied, but the limited evidence suggests significant impacts on an individual's phenotype (Gilad et al. 2018; Paul, Putra, and Müller 2019). Stress experienced during sensitive phases may cause more pronounced changes in phenotypes, since the receptivity might be increased (Cheng et al. 2021). Among ecological stressors, nutritional stress, caused by inadequate food quality or quantity or high density, is a pervasive challenge faced by organisms in natural environments, exerting significant selective pressure on various life‐history traits, including reproduction (Bauerfeind and Fischer 2005; Frago and Bauce 2014; Holmes, Nelson, and Lougheed 2020; Knapp and Uhnavá 2014). Food limitation may invoke complex developmental, behavioural and physiological adjustments aimed at maximising fitness during development (Rashid, Wong, and Roy 2021), enhancing foraging efficiency (Scharf 2016) and prioritising essential physiological processes, such as increased lipid accumulation (Ziegler 1991; Wang et al. 2016; Yamada et al. 2018). Furthermore, a balance between lipids and carbohydrates is important to attain both long‐term and short‐term energy reserves, respectively, particularly under starvation (McCue 2010).

In addition to differences between life stages, organisms often show sex‐specific differences, for example in their physiology, and therefore potentially also in their responses to nutritional stress during sensitive phases. For instance, in *Tribolium castaneum* , adult females are overall more starvation‐tolerant than males when experiencing 4 days of starvation (Gilad et al. 2018). However, in many insect species, early‐life food stress has been found to have a more negative impact on females than males (Teder and Kaasik 2023). Differences in sensitivity between sexes may arise if the timeframes of sensitive phases differ, for example, due to different development times (Rohde, Dreher, and Hochkirch 2015) or sexual size dimorphism (Shingleton and Vea 2023). Sexual size dimorphism may also correlate with sex‐specific plasticity, as found in adult *Drosophila* (Vea et al. 2023), shaping behaviour and metabolic regulation in female and male phenotypes from early life onwards (Shingleton and Vea 2023). The intertwined sex‐specific effects of stress during sensitive phases in insects are still not well understood.

The turnip sawfly, *Athalia rosae* (Hymenoptera: Tenthredinidae), is a holometabolous species characterised by a complex life cycle. The larvae feed on leaves and at later stages also on flowers of Brassicaceae species (Bandeili and Müller 2010), including crops such as cabbage and rapeseed, where they can reach pest status (Saringer 1976). In contrast, the adults feed on nectar from Apiaceae species. Depletion of nutritional resources can occur at different life stages. For example, high numbers of eggs laid on a plant can cause rapid food exhaustion by the hatching larvae (Saringer 1976). Adults may experience nutritional stress due to ephemeral food sources, for example, if they pupated in habitats with suitable food plants that are no longer available at the time of adult emergence. In those cases adult would first need to disperse and may experience nutritional stress until proper habitats are found (Oishi et al. 1993). Starvation during the larval stage has been shown to prolong the development time and decrease adult body mass but had no effects on adult lifespan of *A. rosae* (Paul, Putra, and Müller 2019; Paul et al. 2022). Starved larvae showed increased activity levels, while starved adults showed reduced activity levels compared to non‐starved individuals (Singh et al. 2023). However, little is known about the role of larval versus adult starvation with regard to life stage‐specific sensitivity and trait‐specific responses.

In this study, we investigated the effects of single or repeated nutritional stress events (i.e., starvation) experienced during either the larval and/or adult stage, on life‐history traits, behaviour and metabolic traits of adults, to assess the sensitivity of these life stages and identify which traits are most impacted by such stress. We expected the most pronounced effects on individuals that were starved during both larval and adult stage, but intermediate effects for individuals only starved as larvae or adults. We predicted that larval starvation would prolong development time, reduce body mass and decrease lifespan. With regard to behaviour, we expected starved adults to show lower activity levels, while in terms of metabolism, we anticipated the highest levels of lipids in non‐starved individuals, as they should have been continuously able to build up energy reserves. Repeatedly starved individuals may rely more on carbohydrates as they cannot build up lipids to the same extent; therefore, their lipid contents might be lower. Starvation only during the larval stage may trigger conforming mechanisms, such as an enhanced accumulation of lipids, potentially allowing recovery. Due to sex‐specific differences in the uptake of nutrients (Maklakov et al. 2008) and often more pronounced negative effects of starvation on developmental traits of females in many insect species (Teder and Kaasik 2023), we expected more pronounced effects of early‐life food stress on females than on males. Overall, we aimed to unravel the complex effects of nutritional stress experienced across ontogeny on the outcome of individualised niches.

## 2 Material and Methods

### 2.1 Rearing of Insects and Plants

Adults of *A. rosae* were collected during late summer in the surroundings of Bielefeld, Germany. For a continuous rearing, adults were offered potted plants of white mustard ( *Sinapis alba* , Brassicaceae) for oviposition. After larval hatching, potted, non‐flowering plants of cabbage ( *Brassica rapa* var. *pekinensis*, Brassicaceae) were provided as food source. Both plant species were grown from seeds (KWS Saat; Kiepenkerl, Germany) in a controlled environment (greenhouse or climate chamber, about 20°C, 16L:8D, 70% relative humidity). Insects were kept in cages (60 × 60 × 60 cm) in similar conditions as the plants, but at around 50% relative humidity. For pupation, larvae of the last instar, called eonymphs, went into the soil provided in Petri dishes or in the potting soil. Emerging F1 adult individuals were allowed to mate and provided with 2% honey water. Thirty of these females were set up in a new cage with white mustard for oviposition and removed after 48 h from the cage. After 5 days, 120 freshly hatched larvae (F2) were carefully transferred to individual Petri dishes (5.5 cm diameter) lined with moist filter paper, placed in a climate cabinet under constant conditions (20°C, L16:D8, 65% r.h.) and used for the experiments. The larvae were offered discs (22 mm diameter) of middle‐aged cabbage leaves ad libitum.

### 2.2 Starvation Treatments and Measurements of Life‐History Traits

**FIGURE 1:**
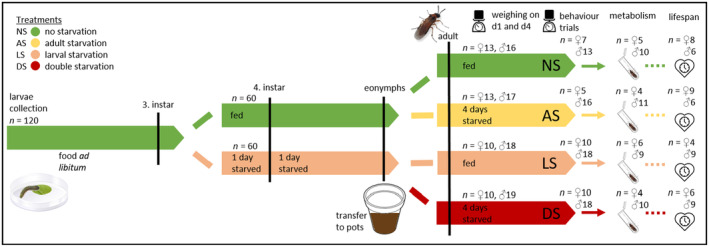
Experimental design for testing sensitive phases to nutritional stress. Individuals of *Athalia rosae* were assigned to different starvation treatments. Data on life‐history (development time, body mass, lifespan) as well as behaviour and metabolic traits of adults were collected.

To study the effects of starvation experience during the larval and/or adult phase on life‐history traits, behaviour and metabolism of *A. rosae* , the following starvation treatments were set up (Figure 1). Half of the larvae (*n* = 60) were randomly assigned to a larval starvation exposure. From those larvae, cabbage leaf discs were removed twice for 24 h each, once after moulting to the third instar and once again after having moulted to the fourth instar, but water was still provided through a moist filter paper. All larvae were checked daily for their status (instar and alive/dead). Once individuals reached the eonymph stage, the date was noted and the eonymphs were transferred into individual plastic cups with a gauze lid, containing around 30 g of soil for pupation. Upon adult eclosion, the date was again noted to calculate the developmental time, the sex determined, the individual weighed (initial adult body mass; *d* = 0.1 mg, Sartorius LA 120 S, Germany) and adults transferred into individual Petri dishes. Half of the individuals that had been provided with food ad libitum and half of those that had been starved as larvae were assigned randomly to an adult starvation exposure. This resulted in four distinct treatments, individuals that were continuously fed as larvae and adults (NS), individuals that only experienced larval starvation (LS), individuals that only experienced adult starvation (AS) and individuals that experienced starvation during both larval and adult stage (repeated, i.e., double starvation, DS). Individuals assigned to an adult starvation exposure were only provided with water on tissue paper for 4 days. The other adults were provided with a mixture of honey and water (1:10) on tissue paper. Since the sex‐ratio was male‐biased, we had in the end more males than females (for exact n see Figure 1). After 4 days of treatment, each individual was weighed again, and the behaviour of a subset of individuals was tracked within the available time constraints (*n* = 5–18 per treatment group and sex; see Behavioural Trials). Following the behavioural trials, half of the individuals of each treatment group (*n* = 4–11 per treatment group and sex) were frozen at −80°C for analysis of the energy metabolism (see Analysis of Energy Metabolism). Individuals in the other half of each treatment group were all provided with the honey water mix ad libitum and checked daily for survivorship to determine their lifespan.

### 2.3 Behavioural Trials

Behavioural observations were conducted in a controlled environment, between 12 and 5 pm at 20°C room temperature. A light source was provided directly above the test arenas, and the order of testing was randomised across treatments. To measure activity levels, adults were transferred with minimal handling to new empty Petri dishes, and six individuals were filmed in parallel for 1 h with an overhead camera (Computar, USA). Their activity was tracked using Ethovision V7 (centre‐point tracked at 1.92 samples/s; Noldus, Netherlands), extracting the distance moved and the time spent being immobile for each individual.

### 2.4 Analysis of Energy Metabolism

As measures of energy metabolism, individual levels of lipids (i.e., unsaturated fatty acids) and carbohydrates were analysed using a modified method by Cuff et al. (2021). Frozen sawflies were lyophilised and the dry mass measured. Lipids were extracted by soaking each sawfly in 0.5 mL of 1:12 chloroform:methanol (chloroform: HPLC grade, AppliChem; methanol: LC–MS grade, Fisher Scientific) for 24 h. Afterwards, 250 μL of the supernatant was taken for later lipid determination. As much of the remaining supernatant as possible was discarded and any residual solvent was allowed to evaporate. Then the samples were soaked in the same solvent for another 24 h, but this time all supernatant was discarded, to ensure any residual lipids were removed from the sample prior to carbohydrate extraction. For the carbohydrate extraction, the sawfly samples were homogenised in a mill at 30 Hz for 30 s, 0.5 mL of 0.1 M NaOH (VWR International) was added to each sample, samples were incubated on a shaker at 80°C and 250 rpm for 30 min, and then removed and left at room temperature overnight. On the next day samples were centrifuged for 10 min at 13,000 rpm and 450 μL of supernatant was used for carbohydrate determination.

For the determination of unsaturated fatty acids, which make up about 50% of the lipids in Hymenoptera (Aguilar 2021), the respective samples (50 μL) were each mixed with 10 μL of sulphuric acid (95%, VWR International) and incubated at 100°C for 10 min. After a 5 min cooldown, 240 μL vanillin in phosphoric acid (17% in ultrapure water, AppliChem) was added to each sample and the absorbance was measured at 490 nm in a microplate reader in a 96‐well plate (Multiskan FC, Thermo Scientific, USA) adapted from Cheng, Zheng, and VanderGheynst (2011). As a standard dilution series for calibration, a cricket oil (Acheta Cricket Oil, Thailand Unique) in 1:12 chloroform:methanol was measured on the same 96‐well plate in eight concentrations.

For carbohydrate determination, sugars were measured and calibrated by trehalose and glycogen, as these are the most abundant sugars in insects (Jiang et al. 2019). The respective samples (40 μL) were each mixed with 160 μL of anthrone reagent (2 mg/mL anthrone (Roth) in 95% sulphuric acid), incubated for 10 min at 92°C and measured at 620 nm (adapted from Dreywood 1946). For carbohydrate calibration, a standard dilution series of 1:1 trehalose:glycogen (trehalose: 98%, Roth; glycogen: AppliChem) in ultrapure water was used. Each 96‐well plate had four solvent blanks and two calibration rows.

### 2.5 Statistical Analysis

Statistical analyses were conducted using the statistical software R, Version 4.1.3 (R Development Core Team 2010). To compare the larval development time and initial adult body mass between the individuals non‐starved and starved as larvae, a Mann–Whitney *U*‐test was used. For comparing adult traits (i.e., body mass change, distance moved, time spent being immobile, lipid and carbohydrate mass) among the four treatment groups, Kruskal–Wallis tests were used, followed by posthoc pairwise comparisons using Dunn's test with Bonferroni correction. Distance moved and time spent being immobile were tested for correlation with a Spearman rank correlation. Survival probability of individuals was plotted with Kaplan–Meier curves using the survival package (Therneau 2024) and an overall log‐rank test was performed, followed by pairwise log‐rank tests with Bonferroni‐Holm correction of *p* values. Graphical visualisations were generated using the ggplot2 (Wickham 2016) multcompView, ggthemes and ggdist packages.

## 3 Results

### 3.1 Larval Starvation Prolonged Larval Development and Reduced Female Initial Adult Mass

**FIGURE 2:**
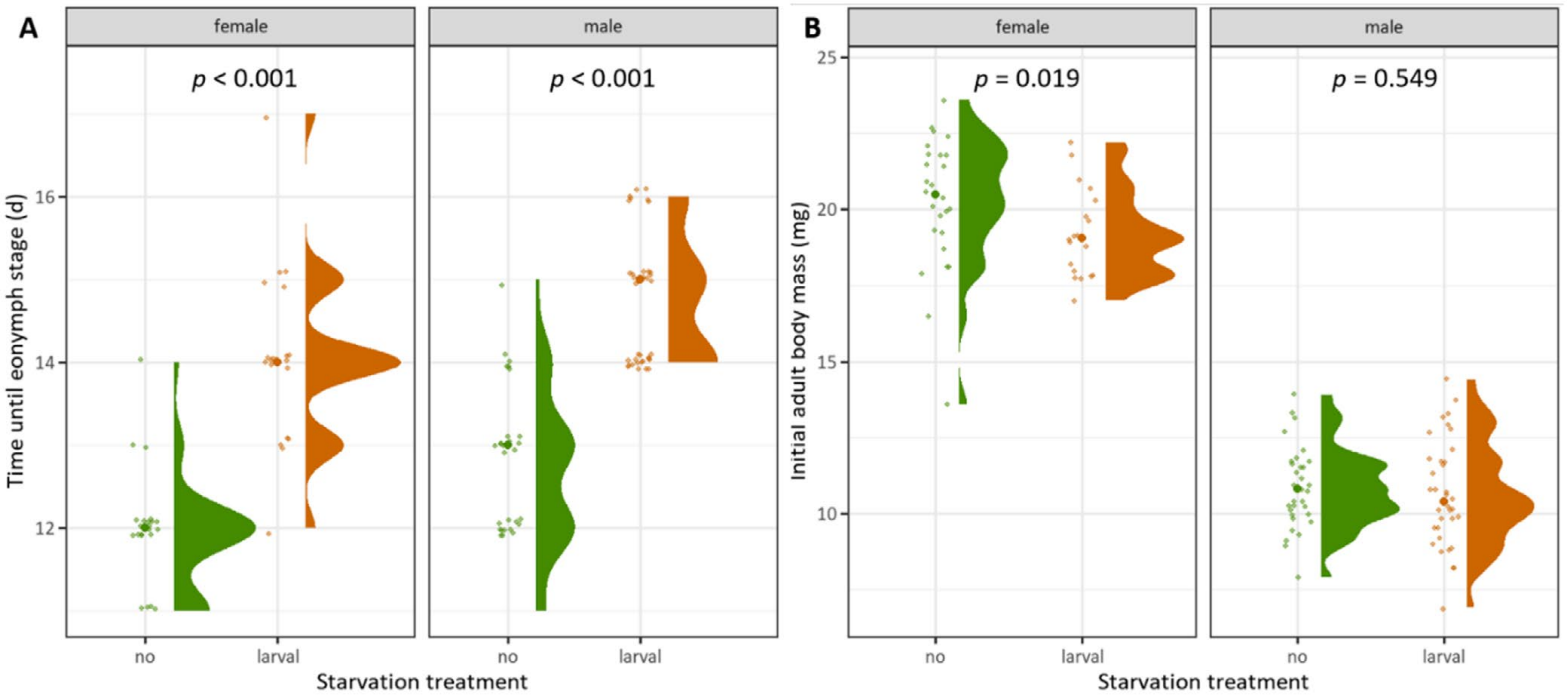
Effect of larval starvation on (A) larval development time until eonymph and (B) initial adult body mass (fresh body mass). Half violin plots indicate the distribution of values, raw data is plotted with slight jittering and thicker dots indicate medians. Statistical differences were tested with Mann–Whitney *U*‐tests, *n* = 10‐19.

Starvation during the larval stage led to a significantly prolonged development for both sexes (females *U* = 24, males *U* = 66; *p* < 0.001), with starved larvae taking nearly 2 days longer than non‐starved ones (Figure 2A). Two females and one male individual from the larval starvation treatment took one additional instar to reach the eonymph stage. The initial adult body mass differed significantly for females (*U* = 366, *p* = 0.019) but not for males (*U* = 662, *p* = 0.549), with females starved as larvae being significantly lighter than non‐starved females (Figure 2B).

### 3.2 Adult and Repeated Starvation Reduced Female Body Mass With No Effect of Starvation Treatments on Lifespan

**FIGURE 3:**
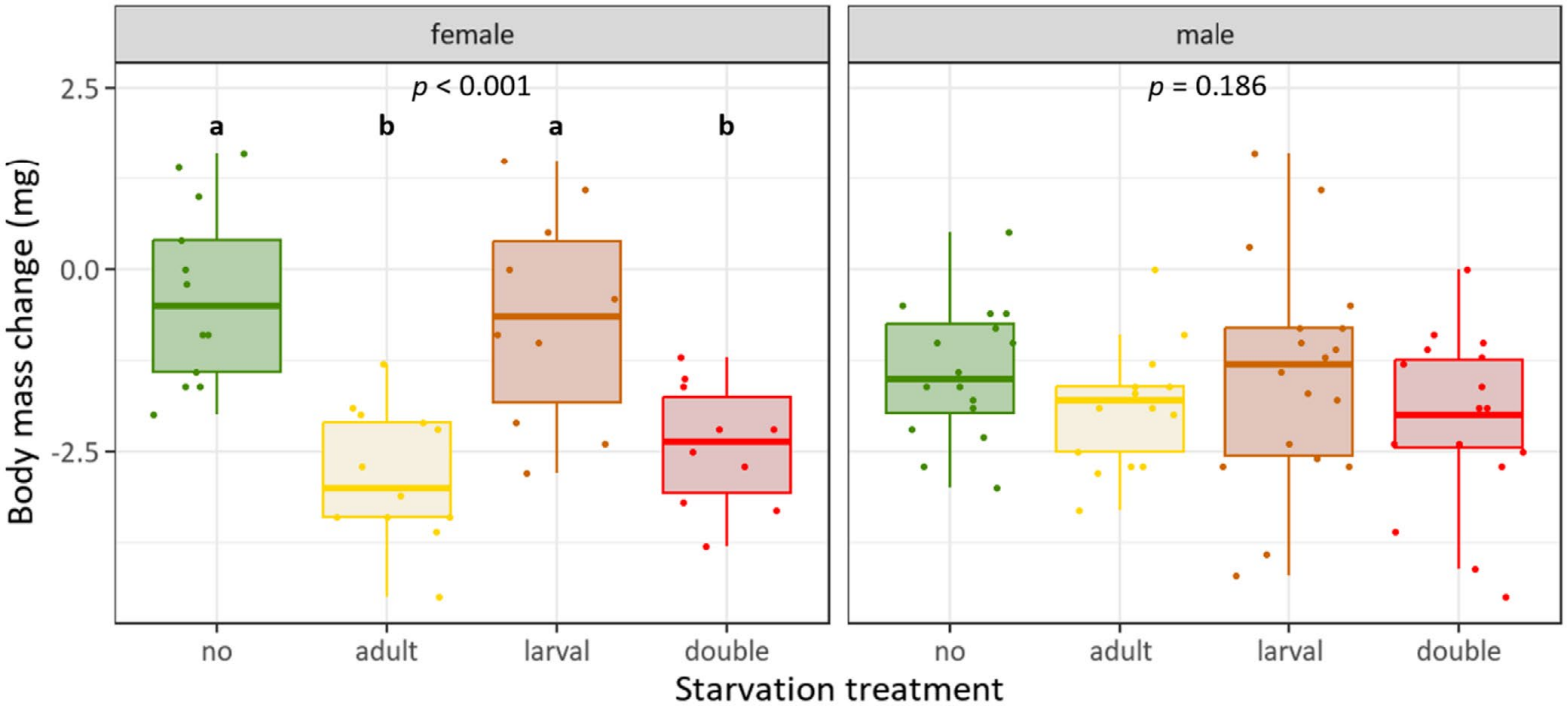
Effects of larval and/or adult starvation on body mass change (fresh body mass) within 4 days in female and male adults. Boxplots indicate interquartile range (IQR, boxes) with medians, whiskers extent to ±IQR*1.5, dots are raw data points. Overall significant differences were tested with Kruskal–Wallis rank sum test, followed by pairwise Dunn posthoc test with Bonferroni correction. Different letters denote significantly different (*p* ≤ 0.05) treatment effects; *n* = 10‐19.

Treatment had a significant effect on adult body mass change in females only (females: *ꭓ* ^2^ = 24.11, *df* = 3, *p* < 0.001; males: *ꭓ* ^2^ = 4.826, *df* = 3, *p* = 0.186). Females starved as adults or as both larvae and adults had a significantly lower body mass change compared to those in the other treatments (Figure 3).

**FIGURE 4:**
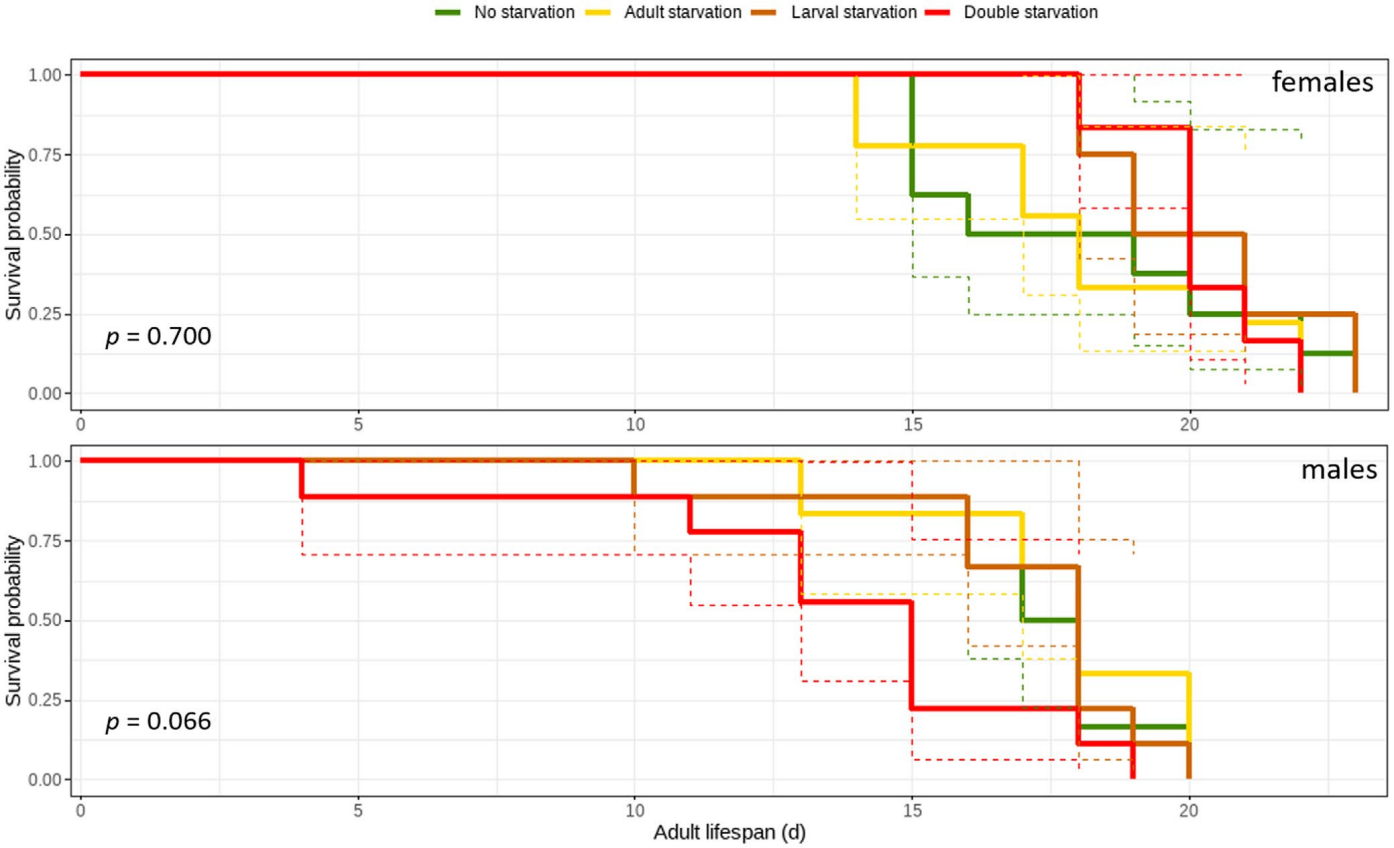
Effect of larval and/or adult starvation on female and male adult survival probability, represented by Kaplan–Meier curves. Dashed lines show 95% confidence intervals. An overall log‐rank test was performed, followed by pairwise log‐rank tests with Bonferroni‐Holm correction of *p* values.

Adult survival probability, that is, lifespan, was not affected by treatment for both females (*ꭓ* ^2^ = 1.4, *df* = 3, *p* = 0.7) and males (*ꭓ* ^2^ = 7.2, *df* = 3, *p* = 0.066). However, males starved at both life stages showed a trend towards lower survival probability (Figure 4).

### 3.3 No Effect of Starvation Treatments on Behaviour

**FIGURE 5:**
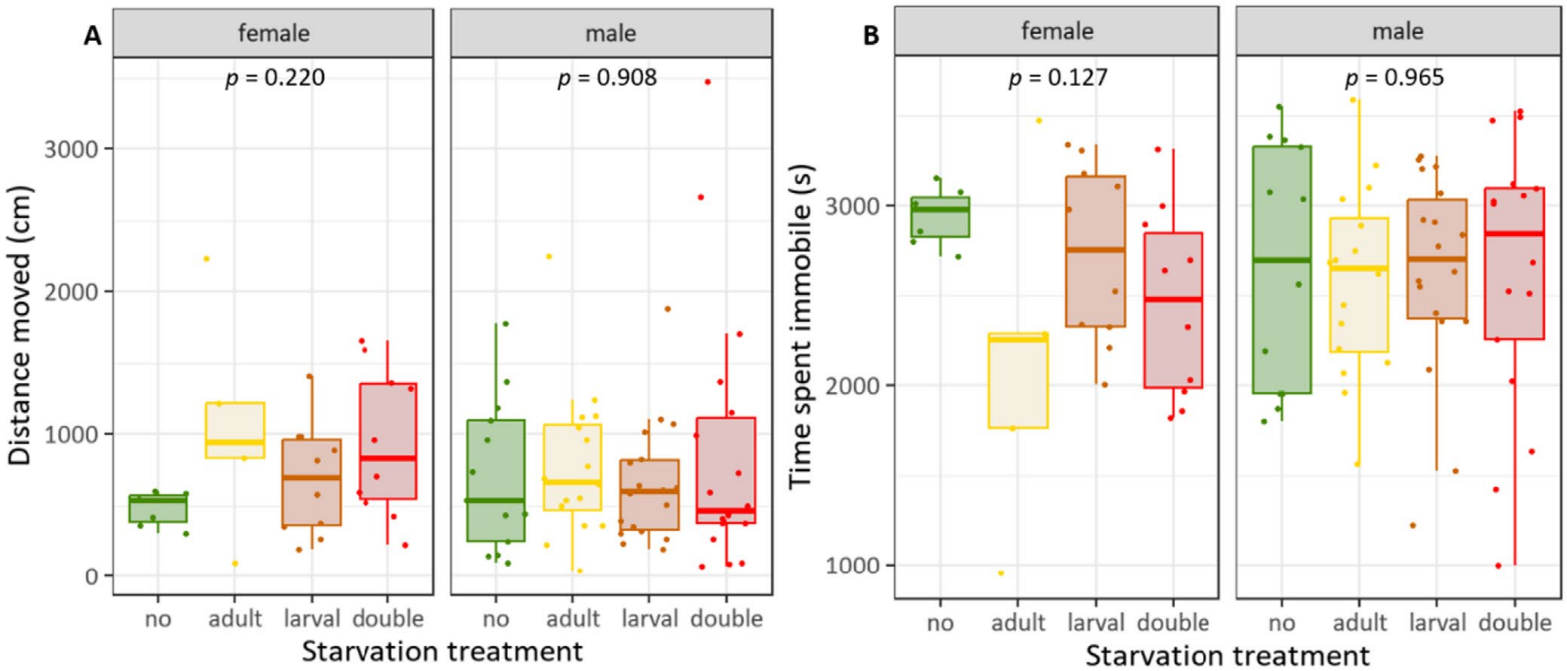
Effects of larval and/or adult starvation treatments on (A) distance moved and (B) time spent being immobile during 1 h of recording of adults. Boxplots indicate interquartile range (IQR, boxes) with medians, whiskers extent to ±IQR*1.5, dots are raw data points. Overall significant differences were tested with Kruskal–Wallis rank sum test, followed by pairwise Dunn posthoc test with Bonferroni correction; *n* = 5‐18.

There was no significant effect of treatment on either distance moved (females: *ꭓ* ^2^ = 4.416, *df* = 3, *p* = 0.220; males: *ꭓ* ^2^ = 0.547, *df* = 3, *p* = 0.908; Figure 5A) or time spent being immobile (females: *ꭓ* ^2^ = 5.702, *df* = 3, *p* = 0.127; males: *ꭓ* ^2^ = 0.273, *df* = 3, *p* = 0.965; Figure 5B) in either sex. The two traits were significantly correlated across all treatments (all *r* ≤ −0.931 and *p* < 0.001).

### 3.4 Adult and Repeated Starvation Led to a Decrease in Both Lipids and Carbohydrates in Males

**FIGURE 6:**
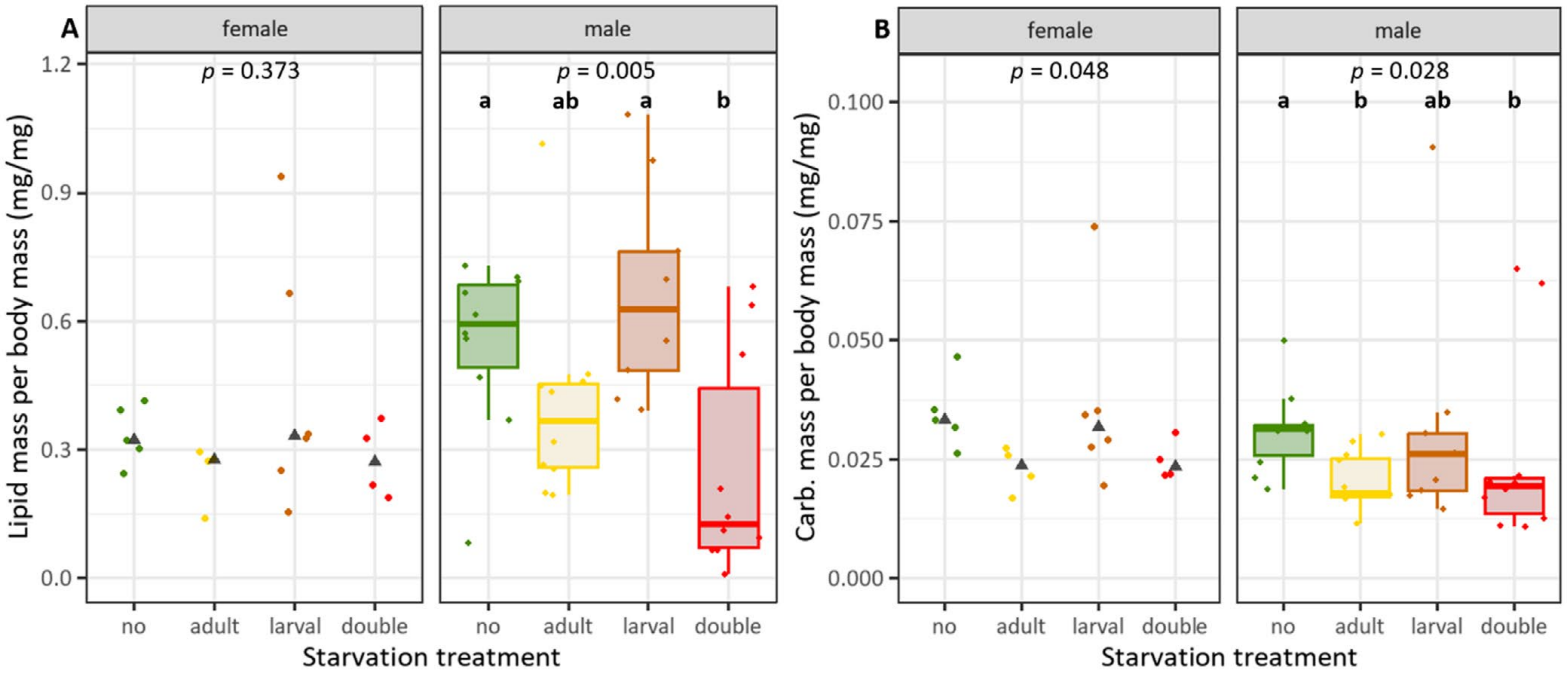
Effects of larval and/or adult starvation treatments on energy metabolism with (A) lipid content per body mass and (B) carbohydrate content per body mass (dry weight) of adult sawflies. Boxplots indicate interquartile range (IQR, boxes) with medians, whiskers extent to ±IQR*1.5, dots are raw data points. Overall significant differences were tested with Kruskal‐Wallis rank sum test, followed by pairwise Dunn post hoc test with Bonferroni correction. Different letters denote significantly different (*p* < 0.05) treatments; *n* = 4‐11. Black triangles indicate the median for females. Boxplots are shown only for males due to *n* = 4 in two female treatment groups.

The lipid mass per body mass was significantly impacted by treatment in males only (females: *ꭓ* ^2^ = 3.122, *df* = 3, *p* = 0.373; males: *ꭓ* ^2^ = 12.793, *df* = 3, *p* = 0.005), with individuals having starved in both the larval and adult stage showing lower lipid amounts compared to non‐starved individuals and to individuals starved only during the larval stage (Figure 6A). The carbohydrate mass per body mass was significantly affected by the starvation treatments in both sexes (females: *ꭓ* ^2^ = 7.908, *df* = 3, *p* = 0.048; males: *ꭓ* ^2^ = 9.137, *df* = 3, *p* = 0.028), although post hoc comparisons showed no significant differences between treatments for females (Figure 6B). In males, those starved as adults or both as larvae and adults had lower carbohydrate levels compared to non‐starved individuals or those starved only during the larval stage.

## 4 Discussion

By recording a comprehensive set of life‐history, behavioural and metabolic traits, we aimed to identify phases during which individuals are most sensitive to starvation. Our findings show that these phases may differ depending on the respective trait.

Starvation during the larval stage led to prolonged development until reaching the eonymph stage and also sometimes additional larval instars. Similar effects were also found in response to different starvation regimes, partly with more frequent starvation events during the larval stage, in the same species (Paul, Putra, and Müller 2019; Paul et al. 2022). With an additional instar, individuals may compensate for inadequate nutrition and reach a larger adult body mass at the expense of a prolonged larval development (Esperk et al. 2007). Delayed development may result from the lack of needed nutrients and is probably also connected to delayed growth hormone production (Friend 1958; Clark and Gibbs 2023). It is well‐documented across many insect taxa that juvenile/larvae exposed to lower resource levels significantly prolong their developmental time and also remain smaller compared to conspecifics in more favourable conditions (Teder, Vellau, and Tammaru 2014). Moreover, consistent with our expectations and findings from other insect species (Teder and Kaasik 2023), we found that only adult females were lighter at eclosion as a result of larval starvation. In numerous insect species with sex dimorphism, males were found to be less affected by adverse environmental conditions than females, tending to lead to reduced sex dimorphism (Teder and Tammaru 2005). Interestingly, male sawflies seemed to be able to compensate for the poor larval conditions, reaching comparable adult body mass as non‐starved larvae. This sex‐specific difference in adult body mass in response to larval starvation is also in accordance with previous studies on *A. rosae* in which larval starvation was performed in a similar regime (Paul et al. 2022). The development of female reproductive organs might be more demanding and prioritised, restricting nutrient availability for biomass production (Jensen et al. 2015). Males may compensate the effect on this trait well by prioritising essential cells and tissue (Metcalfe and Monaghan 2001). However, there are also other studies, which found that females of holometabolous insects may be more plastic in their body size than males (Rohner et al. 2018).

When adults of *A. rosae* were exposed to starvation, again only females showed a significant loss in body mass, with no signs of an additive effect of larval and adult starvation. Rather, the body mass loss was similar for individuals of the doubled starvation treatment compared to individuals that were only starved as adults, which may indicate that individuals that experienced starvation already during early life may have adjusted their metabolism to starvation. These individuals may gain improved digestive efficiency, as has been found in *Drosophila* after experiencing one or more starvation events (McCue, Terblanche, and Benoit 2017). Adult lifespan of *A. rosae* was not affected by any of the starvation treatments, indicating that this species is quite robust even to repeated starvation events as implemented in our study. However, males starved as both larvae and adults showed the lowest lifespan, though this was not statistically significant. Individuals may show different insulin‐signalling and nutrient allocation, as demonstrated in *Drosophila* in response to different dietary restriction regimes experienced as adults, and therefore react with different sensitivity in terms of lifespan (Magwere, Chapman, and Partridge 2004). In conclusion, certain traits may be more affected and sensitive to starvation during specific life stages than others. Especially in a complex interplay with other abiotic factors, consequences on life‐history traits may differ across life stages (Vasudeva 2023). Moreover, the importance of adult feeding differs among insect species; the adults of some species, also called capital breeders, do not feed at all as adults and rely solely on food intake during the larval stage. In other species, called income breeders, the adults feed mainly on carbohydrates and need those resources for reproduction (Tammaru and Haukioja 1996). Several species are in between these extremes, including Hymenoptera (Casas et al. 2005). Adults of *A. rosae* need to take up carbohydrates at some point but can already lay eggs one day after adult emergence, using thus also resources collected as larvae (Sawa et al. 1989). Plasticity may help the larvae to adapt their metabolism and cope better with subsequent nutritional stress, while adult body mass may respond more sensitively to starvation during the adult stage.

In contrast to our expectation, neither larval nor adult starvation affected the activity of the adults, measured as distance moved and time spent being immobile. These two traits were negatively correlated. Different effects of starvation on arthropod behaviour have been reported, ranging, for example, from predation avoidance to increased foraging (Scharf 2016). The life stage at which starvation occurs combined with the chosen behavioural tests can be important factors (Romero, Potter, and Haynes 2010). During early life starvation, insects may increase their activity in order to locate new food sources. In *Drosophila melanogaster* , such hyperactivity has been found to be induced by octopamine after 24 h of sucrose deprivation (Yang et al. 2015). At a more severe level of starvation, activity may be reduced to conserve energy or due to exhaustion (Scharf 2016). We did not test for flight capacity, but inadequate nutrition may reduce flight capacity by causing changes in muscle molecular composition (Portman et al. 2015). Activity‐related behaviours seemed to be less plastic, possibly to maintain key survival functions. This is in contrast to a previous study, in which significant impacts of a four day starvation period on activity of *A. rosae* were found (Singh et al. 2023). Different outcomes may result from slight differences in the setup, such as keeping individuals in groups (Singh et al. 2023) versus keeping them individually (present study) during the rearing phase. Nevertheless, we cannot exclude that longer starvation periods may have led to more drastic changes in activity and that other behaviours, that is, those related to boldness, may be more responsive to the starvation regimes.

In our energy metabolism assay the sex ratio was male‐biased, so we mainly discuss the effects on males, although the observed pattern was similar for both sexes. Lipid levels significantly differed between males from the distinct treatments. Males with no starvation and those exposed to larval starvation showed higher amounts of lipids per body mass than those in the other two treatments, as hypothesised. Upon larval starvation, individuals may build up an enhanced lipid storage. In the grasshopper *Schistocerca americana* , larval nutrition has been found to be particularly relevant for adult lipid storage, with lipid stores being more important than carbohydrate or protein stores (Hahn 2005). In *A. rosae* , the carbohydrate levels showed a somewhat similar pattern to the lipids in response to the treatments, indicating that both energy sources may be equally important for the adults. The mobilisation of more than just one energy source is essential to cope with nutritional stress (Sanathoibi and Keshan 2019). For instance, honey bees starved as larvae improve their metabolic response to adult starvation mainly through carbohydrates (Wang et al. 2016). Different species may thus preferably use different energy sources. Larval starvation alone may lead to niche conformance, with individuals building up energy reserves, whereas adult starvation and repeated starvation in the larval and adult stages may lead to an increased depletion of both energy sources. Sensitive phases in insects, such as the larval stage with higher plasticity, can have lasting impacts on the adult physiology and the ability to manage energy resources. In a world with global climate change, life‐history traits might be impacted through physiological shifts, and it becomes even more important to understand the role of sensitive phases, plasticity and the ability to conform.

## 5 Conclusion

Our study revealed life stage‐ and sex‐specific effects of nutritional stress on various parameters involving life‐history traits, behaviour and metabolism. The effects differed across the recorded traits. Life‐history traits were affected most significantly, especially in females. Activity‐related behaviour seemed to be a more conserved trait in our study, while energy metabolism was mainly affected by adult starvation and starvation during the larval and adult stages. Our findings indicate that the sensitivity of a certain life stage to nutritional stress depends on the specific trait under consideration and is not uniform across all traits in a single life stage. The larval stage appears more sensitive with respect to life‐history traits, whereas the adult stage seems more sensitive in terms of metabolism. Additionally, the intensity and frequency of stressors are decisive. Being sensitive with regard to specific traits in different stages may lead to distinct individualised niches.

## Author Contributions

**Leon Brueggemann:** conceptualization (equal), data curation (equal), formal analysis (equal), investigation (equal), methodology (equal), software (equal), validation (equal), visualization (equal), writing – original draft (equal). **Pragya Singh:** conceptualization (equal), methodology (equal), software (equal), supervision (equal), validation (equal), writing – review and editing (equal). **Caroline Müller:** conceptualization (equal), data curation (equal), funding acquisition (equal), project administration (equal), resources (equal), supervision (equal), validation (equal), writing – review and editing (equal).

## Conflicts of Interest

The authors declare no conflicts of interest.

## Data Availability

Data are available via gitlab. https://gitlab.ub.uni‐bielefeld.de/lbrueggemann/nutritional‐stress‐in‐sensitive‐phases.
